# piRNAQuest: searching the piRNAome for silencers

**DOI:** 10.1186/1471-2164-15-555

**Published:** 2014-07-04

**Authors:** Arijita Sarkar, Ranjan Kumar Maji, Sudipto Saha, Zhumur Ghosh

**Affiliations:** 1 Bioinformatics Centre, Bose Institute, Kolkata 700054, India

**Keywords:** piRNA, Repeat element, Cluster, Transposable element, Motif, Stem cells, Spermatogenesis

## Abstract

**Background:**

PIWI-interacting RNA (piRNA) is a novel and emerging class of small non-coding RNA (sncRNA). Ranging in length from 26-32 nucleotides, this sncRNA is a potent player in guiding the vital regulatory processes within a cellular system. Inspite of having such a wide role within cellular systems, piRNAs are not well organized and classified, so that a researcher can pool out the biologically relevant information concerning this class.

**Description:**

Here we present piRNAQuest- a unified and comprehensive database of 41749 human, 890078 mouse and 66758 rat piRNAs obtained from NCBI and different small RNA sequence experiments. This database provides piRNA annotation based on their localization in gene, intron, intergenic, CDS, 5^/^UTR, 3^/^UTR and repetitive regions which has not been done so far. We have also annotated piRNA clusters and have elucidated characteristic motifs within them. We have looked for the presence of piRNAs and piRNA clusters in pseudogenes, which are known to regulate the expression of protein coding transcripts by generating small RNAs. All these will help researchers progress towards solving the unanswered queries on piRNA biogenesis and their mode of action. Further, expression profile for piRNA in different tissues and from different developmental stages has been provided. In addition, we have provided several tools like 'homology search’, 'dynamic cluster search’ and 'pattern search’. Overall, piRNAQuest will serve as a useful resource for exploring human, mouse and rat piRNAome. The database is freely accessible and available at http://bicresources.jcbose.ac.in/zhumur/pirnaquest/.

**Conclusion:**

piRNAs play a remarkable role in stem cell self-renewal and various vital processes of developmental biology. Although researchers are mining different features on piRNAs, the exact regulatory mechanism is still fuzzy. Thus, understanding the true potential of these small regulatory molecules with respect to their origin, localization and mode of biogenesis is crucial. piRNAQuest will provide us with a better insight on piRNA origin and function which will help to explore the true potential of these sncRNAs.

## Background

Piwi-interacting RNAs (piRNAs) constitutes a novel class of small ncRNA [1,2] which was first reported in mouse testes in 2006 simultaneously by several research groups [3-7]. These ncRNAs are 26-32 nucleotides (nts) in length and bind to PIWI family of proteins (PIWI, MIWI, MILI, HIWI, HILI). As PIWI proteins are mostly confined to germline cells and stem cells, piRNAs are abundant in spermatogenic cells and are responsible for stem cell self-renewal [8]. These versatile small molecules have a wide role in cellular system. They are associated with silencing of transposons thereby protecting the genome from the activity of 'parasitic nucleic acid’ [9]. Epigenetic inactivation of the PIWI pathway genes produces defective piRNAs. This leads to an imbalance in methylation which contributes to an unsuccessful germ cell development and results in male infertility disorders [10]. Moreover, spermatocytes deficient in piRNAs during the pachytene stage become arrested at the post-meiotic round spermatid stage with massive DNA damage [11]. Apart from such role in developmental biology, this class of small ncRNAs (sncRNAs) have a predominant role within cancer cells. PIWI orthologs such as HIWI, HILI, are reported to be overexpressed in a variety of human cancers [12,13].

### Origin of piRNAs, their biogenesis and function

With the advent of new technologies achieving unprecedented depths in RNA sequencing, several thousands of piRNAs have been identified across the mammalian genome with diverse genomic context and mechanistic details [14]. These map to various regions of the genome (5^/^UTR, 3^/^UTR, CDS, intron and intergenic) [15]. The biogenesis of piRNAs is thought to be mediated by 'Ping-Pong’ amplification cycle [16]. But the exact mechanism of their biogenesis is still not clear. Further, it has been observed that the mode of piRNA biogenesis varies, depending on its genomic location [17]. Of all piRNAs, intergenic piRNAs have been widely studied. Some intergenic piRNAs are devoid of transposable/repetitive elements and follow a mode of biogenesis that might differ from 'Ping-Pong’ amplification cycle [15,17]. These piRNAs, in large numbers are found in meiosis and post-meiotic stages and it is during these stages that the chromosomes are remodelled [15]. It is hence assumed that intergenic piRNAs might be involved in chromosome remodelling. Thus, studying intergenic piRNAs will help us understand the mode of biogenesis which is unlike the 'Ping-Pong’ cycle.

Apart from these, piRNAs originate from pseudogenes [15]. Pseudogenes are the non-functional counterpart of protein coding genes. Initially these were thought to be 'junk DNA’, but on further exploration it was observed that these too harbours ncRNA. Several sncRNAs like small interfering RNAs (siRNAs), microRNAs (miRNAs) have been reported to be generated from pseudogenes and these in turn regulates the expression of protein-coding genes [18]. Thus pseudogenes are capable of regulating the protein-coding genes with the help of sncRNAs.

piRNAs mostly arise from clusters within the genome [5]. These regions are the 'hotspots’ of biogenesis which synthesizes multiple piRNAs from a particular stretch of genomic loci. These hotspots are either mono-directional (wherein piRNAs are derived from either plus or minus strand) or bi-directional (wherein piRNAs are derived from both strands). Bi-directional clusters have two transcripts and are divergently transcribed from a common promoter [17]. piRNA clusters are conserved in the syntenic regions of a few mammalian species like human, mouse and rat [5,19]. piRNAs mostly arise from transposable elements of the genome and they safeguard the genome by silencing these transposons [9] which contains repeat elements. On the contrary, mouse testes also constitute several piRNAs that map to non-repetitive elements [3,5,7]. It has been reported that most piRNAs of meiotic spermatocyte are derived from non-repeat intergenic regions [20]. Thus, this class of sncRNA is versatile and plays a major role in regulating vital processes within cellular system.

### Existing databases and their pitfalls

Inspite of having such a wide distribution and role within cellular system, this new class of sncRNA has not been organized in a manner by which a researcher can pool out the biologically relevant information regarding them. Existing databases like piRNABank [21], NONCODE [22], RNAdb [23], piRNA cluster database [24], deepBase [25] and GeneCards [26] have provided information on piRNAs. piRNABank [21] has mapped the piRNAs to chromosomal locations of the reference genome, which provides visualization of piRNAs overlapping a specific gene and has annotated piRNA clusters. NONCODE [22] lists the description of piRNAs like length, sequence, cellular location, CPC score of the piRNA. RNAdb [23] provides only the sequence of piRNAs. piRNA cluster database enlists the clusters determined for some species using proTRAC [24]. DeepBase [25] provides the genomic locations of repeat-associated small interfering RNAs (rasiRNAs). GeneCards [26] gives only the genomic loci for the piRNA. Despite this, there still remain open untrodden avenues of analyzing piRNA biology and function: distribution of piRNAs within gene, intron, intergenic, CDS, 5^/^UTR, 3^/^UTR, pseudogenes and repeat elements, variation in their mode of biogenesis and functions; distribution of piRNA clusters, their characteristic motifs and association with long noncoding RNAs (lncRNAs); tissue and stage-specific expression pattern of piRNAs. These are crucial features to consider while unravelling the functional complexity of piRNAome. Such incompleteness in existing information on piRNAs motivated us to analyze these aspects of piRNA and develop piRNAQuest. piRNAQuest provides an extensive categorization of human, mouse and rat piRNAs obtained on analysing experiments reported till date in NCBI [27] (GenBank [28], GEO [29] and SRA [http://www.ncbi.nlm.nih.gov/sra]) as well as supplementary information of the studies. piRNA annotation has been done based on their localization within gene, intron, intergenic, CDS, 5^/^UTR, 3^/^UTR, and repeat elements. We have also looked into the possible Ping-Pong features of the piRNAs (Ping-Pong piRNAs) such as bias for 5^/^-Uracil in the first nucleotide and presence of adenine at the 10^th^nucleotide from the 5^/^-end. Moreover, we have annotated the Ping-Pong partners based on 10 nucleotide overlap between Ping-Pong piRNAs. We have also annotated piRNA clusters for human, mouse and rat respectively. Further, we have elucidated the *characteristic motifs* of the piRNA clusters which might provide a clue regarding the target binding specificity of these clusters. We have also calculated GC content of both piRNA clusters and piRNA sequences which can provide an overview on the target binding stability of the piRNAs. We have curated the putative promoter regions for piRNA clusters and have also looked for the presence of piRNAs and piRNA clusters within pseudogenes. To check evolution of the piRNA clusters and its constituent piRNAs across species, we have mapped piRNA loci with the syntenic regions between human, mouse and rat genome. We have provided tissue and stage-specific expression profile of piRNAs based on the small RNA sequencing experiments reported till date in NCBI (GEO, SRA) as well as supplementary information of the studies.

An added feature of our database are the tools wherein users would be able to search homology with our annotated piRNAs, a dynamic cluster determination tool, using which users can redefine one’s own piRNA cluster. Pattern search has been provided to help users search for any motif within the piRNAs present in our database and the detailed information regarding them. AT-GC percentage calculator has also been added to check the nucleotide content of any queried sequence.

Overall, piRNAQuest will serve as a useful resource for both computational and experimental biologists to browse, search and retrieve information on human, mouse and rat piRNAs.

## Construction and content

### Annotating genomic location of piRNAs

Small RNA read sequences for human, mouse and rat was obtained from experiments reported in (GEO, SRA) as well as from supplementary information of the studies (given in Additional file 1: Table S1). The sequences for a particular organism were then aligned with the reference genome. The unaligned reads and those mapping to other ncRNAs (miRBase miRNAs, Rfam: rRNA, tRNA, snoRNA) were filtered out. The remaining mapped reads were used to predict novel piRNAs using piRNApredictor [30]. These predicted piRNA sequences obtained from experiments (GEO, SRA), sequences submitted in GenBank and other reported piRNAs in literatures were collated. The non-redundant sequences were filtered thereafter and unique piRNAQuest IDs were allocated to them. These piRNAQuest piRNAs were then used for further classifications (Additional file 2: Figure S1). 41749, 890078 and 66758 piRNA sequences for human, mouse and rat respectively were annotated. Among the annotated piRNAs, 68% in human, 75% in mouse and 76% in rat uniquely mapped to the genome. Human, mouse and rat genome sequences (in *.fasta* format) corresponding to hg19/Build37.3, mm10/Build 38.1 and rn5.0/build5 respectively were obtained from NCBI. We have used a nomenclature to designate the annotated piRNAs. piRNAQuest uses a three letter abbreviated prefix to designate the organism (hsa: Human, mmu: Mouse and rat: Rat), followed by the abbreviation 'piRNA’ and an unique identifier for each piRNA sequence. Thus the piRNA identifier takes the form '*hsa_piRNA_6754'* for piRNAQuest annotated human piRNA.

**Figure 1:**
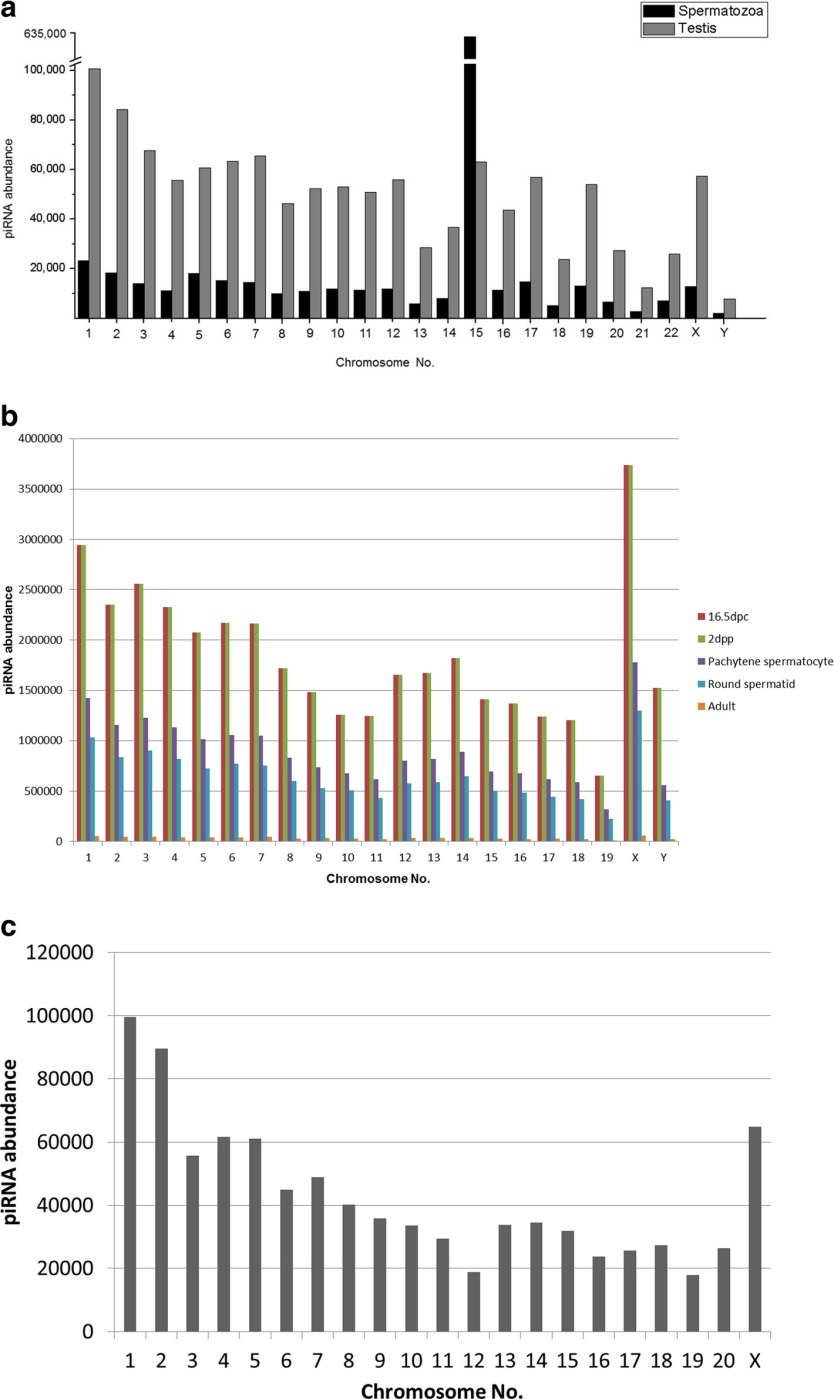
Distribution of (a) human piRNAs across all chromosomes in spermatozoa and testes, (b) mouse piRNAs across all chromosomes in 16.5dpc, 2dpp, pachytene spermatocyte, round spermatid and adult stage and (c) rat piRNAs in testes.

We aligned the annotated piRNA sequences with the corresponding human, mouse and rat genome using BLAST [31]. Default parameters were used for the alignment. Alignments with no mismatch, no gaps, match length = query length were considered for further analysis. An e-value cut-off of 10^-7^ was further imposed on the output to obtain the significant alignments within the genome. On imposing these filters we obtained 804311, 56062307 and 904717 alignments in human, mouse and rat respectively. Figure 1 shows the distribution of piRNAs across all chromosomes in human, mouse and rat genome. As can be observed from Figure 1, spermatozoa-derived and testes-derived human piRNAs show maximum abundance in chromosome 15 and 1 respectively. For mouse, piRNAs from 16.5dpc, 2dpp, pachytene spermatocyte, round spermatid, and adult stages shows maximum abundance in chromosome X whereas, chromosome 1 in rat testes shows maximum piRNA abundance.

### Classifying the annotated piRNAs

As piRNAs are smaller, about 26-32 nucleotides in length, so, they are widely distributed throughout the genome. Since the mode of piRNA biogenesis varies, depending on its genomic location [15,17], it is important to study the distribution of piRNAs across the entire human, mouse and rat genome. Chromosomal positions corresponding to genes, introns, CDS, 5^/^UTR, 3^/^UTR, for human, mouse and rat genome were downloaded from UCSC genome browser [32]. Using in-house perl scripts, we determined the localization of the piRNAs within the genes, introns, intergenic regions, CDS, 5^/^UTR, 3^/^UTR using the corresponding coordinate information from UCSC genome browser.

**Figure 2:**
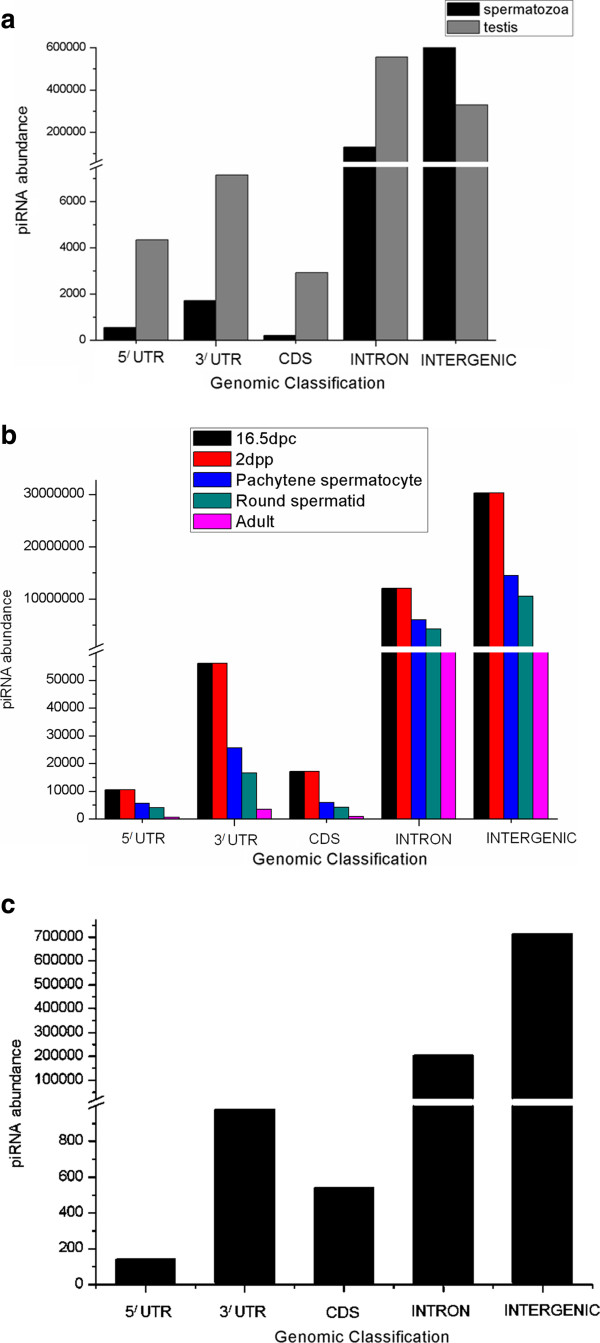
**Distribution of piRNAs across 5**^**/**^**UTR, 3**^**/**^**UTR, CDS, intron, intergenic regions in: (a) spermatozoa and testes of human, (b) 16.5dpc, 2dpp, pachytene spermatocyte, round spermatid and adult stage of mouse and (c) testes of rat.**

Figure 2 shows piRNAs overlapping with intron (intronic piRNAs), intergenic (intergenic piRNAs), 5^/^UTR (5^/^UTR piRNAs), CDS (CDS piRNAs) and 3^/^UTR (3^/^UTR piRNAs) in human, mouse and rat. Our observation reveals that there is a non-uniform distribution of human, mouse and rat piRNAs across different genomic locations as was also concluded by Gan et al. [15] from his investigation with the piRNAs involved in different stages of spermatogenesis. Further, there is a prevalence of intronic and intergenic piRNAs in human, mouse and rat.

### Distribution of repetitive elements in piRNAs

piRNAs are known to be mostly derived from the repetitive regions of the genome and they safeguard the genome by targeting transposable elements (containing repeats) [14]. We thus investigated (a) *the localization of piRNAs within repeat regions* and (b) *searched for the presence of various repeat elements within piRNAs*. This information will help users explore whether the various types of repeat within the piRNAs influence the mode of regulation [14] imposed by piRNAs in guiding the vital pathways of a cell including germline development.

**Figure 3:**
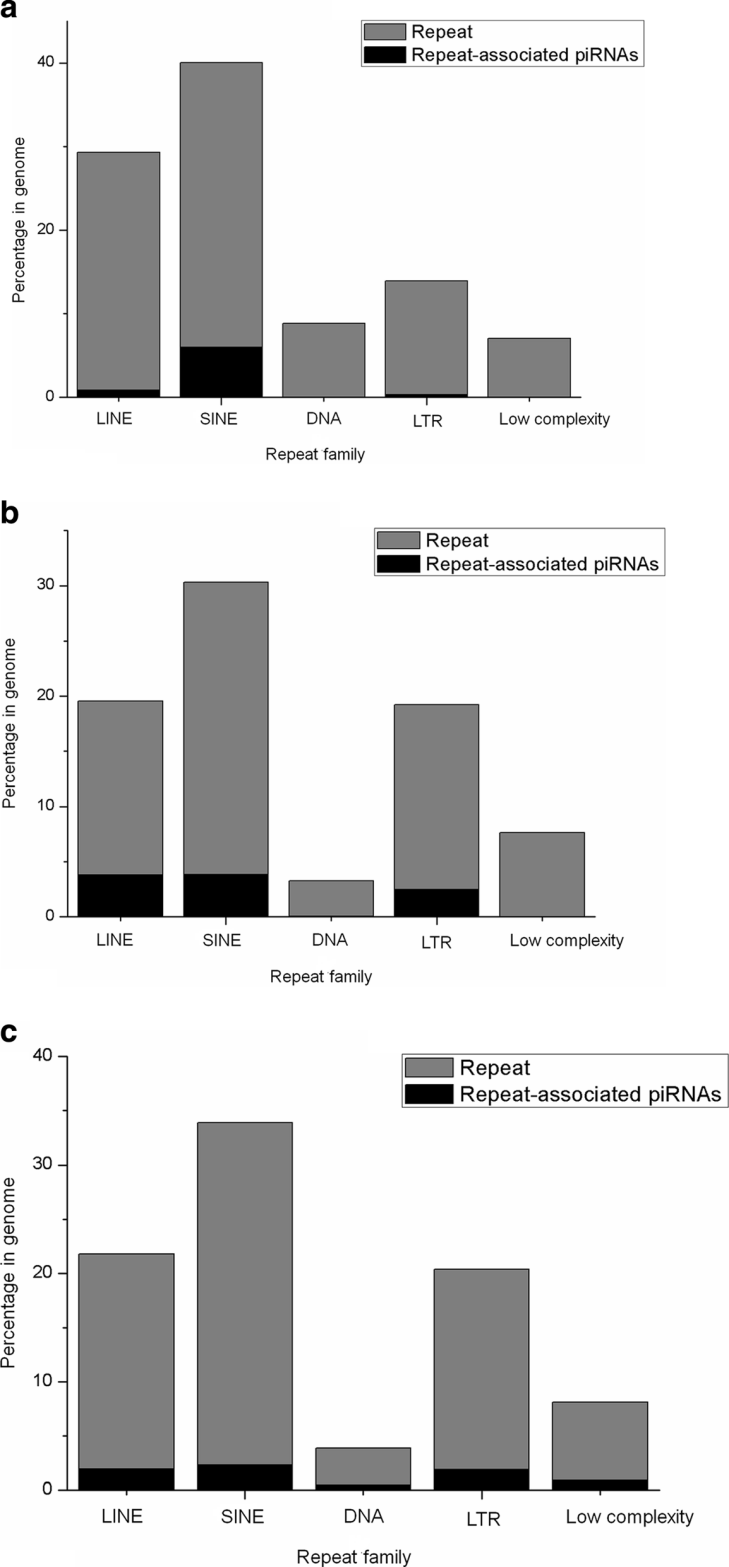
Bar-chart representing the localization of repeat-associated piRNAs within different repeat families across (a) human genome (b) mouse genome and (c) rat genome.

**Figure 4:**
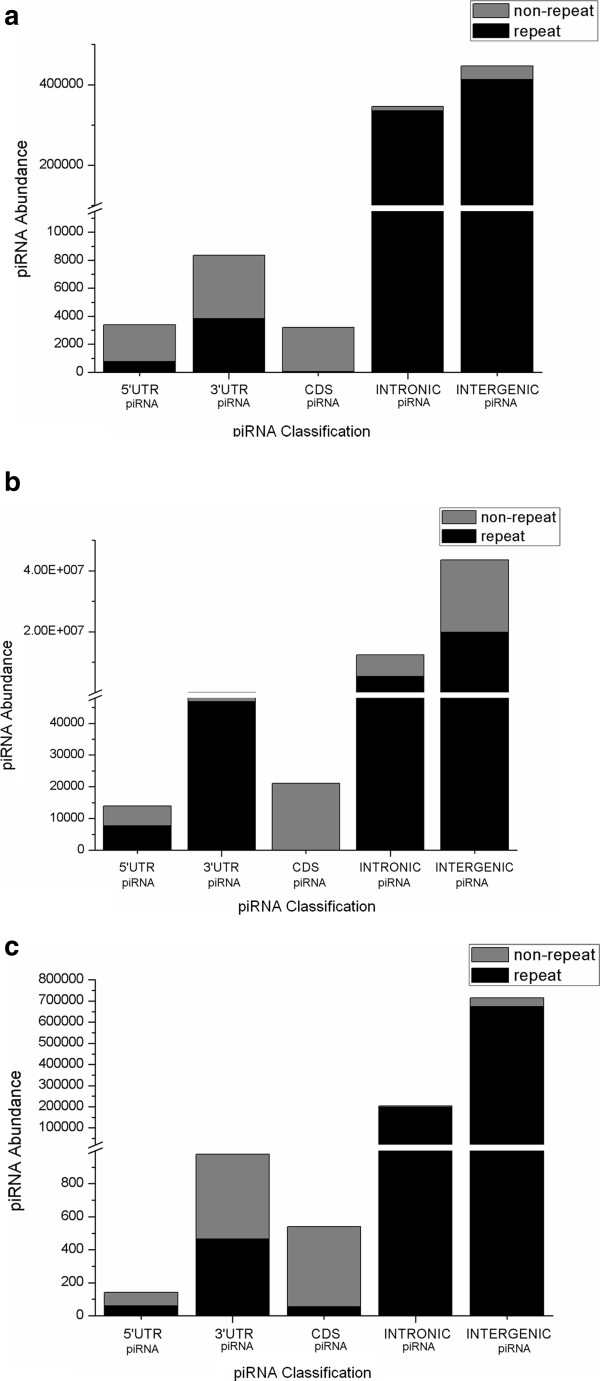
**Distribution of repetitive and non-repetitive elements in 5**^**/**^**UTR piRNAs, 3**^**/**^**UTR piRNAs, CDS piRNAs, intronic piRNAs and intergenic piRNAs for (a) human genome (b) mouse genome and (c) rat genome.**

(a) To study *the localization of piRNAs within various repeat families*, we mapped the genomic coordinates of the piRNAs (obtained from BLAST) with the chromosomal positions corresponding to the repetitive regions in human, mouse and rat genome downloaded from UCSC genome browser [32]. The result is given in Figure 3 which shows a preponderance of the SINE associated piRNAs followed by LINE and LTR associated piRNAs in human, mouse and rat genome.

(b) To *search for the presence of various repeat elements within piRNAs*, we checked the distribution of repetitive elements in 5^/^UTR piRNAs, 3^/^UTR piRNAs, CDS piRNAs, intronic piRNAs and intergenic piRNAs. In case of human, mouse and rat, majority of intronic and intergenic piRNAs comprised of repetitive elements, a major portion of CDS piRNAs comprises of non- repetitive element as shown in Figure 4. These non-repetitive piRNAs in human, mouse and rat might follow a mode of biogenesis which is unlike the 'Ping-Pong’ cycle [17].

### Annotating piRNA clusters - the 'hotspots’ of piRNA biogenesis

It has been reported in literature that piRNAs form clusters i.e. these are 'hotspots’ within the genome which gives rise to a bulk of piRNAs. We identified the piRNA clusters, based on the definition of Lau et al. [4]. Clusters were identified by scanning windows of 20 kilobases (kb) with an interval of 1 kb across the chromosome and detecting those genomic regions with density of ~20-50 piRNAs. We varied the piRNA density on the basis of distribution of piRNAs across different organisms. On encountering such a window, the right hand boundary of the window was extended progressively by 1 kb till the density drops below ~20-50. Within this window, the genomic position of first nucleotide of the first piRNA hit and genomic position of the last nucleotide of the last piRNA hit was considered as the boundaries of the piRNA cluster. Based on this definition, in-house perl scripts were developed to annotate human, mouse and rat piRNA clusters. We further assigned score to each of the clusters based on piRNA density within a cluster, cluster length and window length [33]. The cluster score is calculated as:

$$\mathrm{Cluster}\;\mathrm{Scor}e_{i}=\frac{\frac{\mathrm{piRNA}\;\mathrm{count}\;{\left(\mathrm{in}\;1000\;\mathrm{Kb}\right)}_{i}}{\mathrm{cluster}\;\mathrm{lengt}h_{i}}}{\frac{\mathrm{piRNA}\;\mathrm{density}}{\mathrm{window}\;\mathrm{length}}}$$

A higher cluster score for a cluster, implies greater density of piRNAs within the cluster.

**Figure 5:**
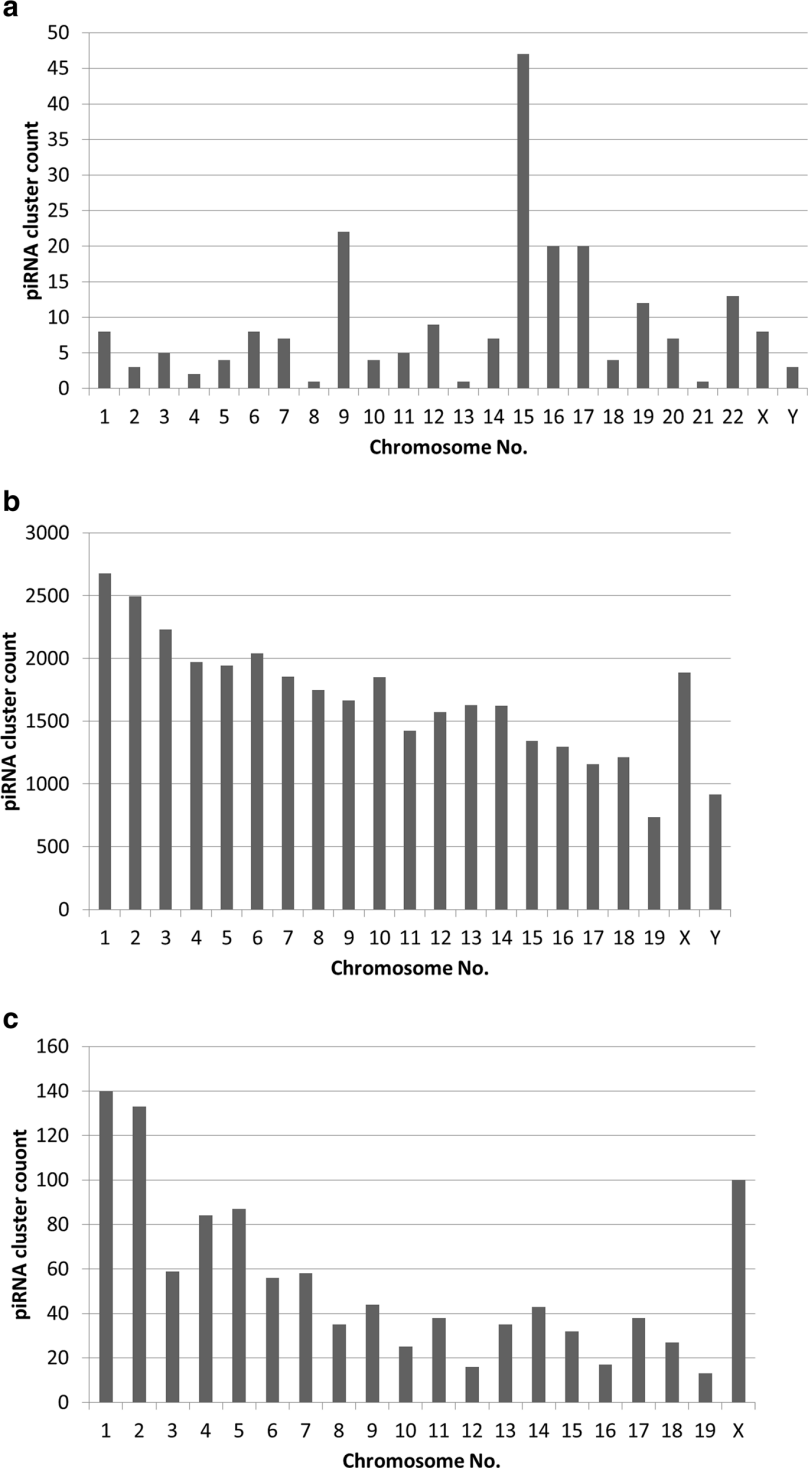
piRNA clusters across the entire (a) human genome (b) mouse genome and (c) rat genome.

We annotated 221, 35250 and 1080 piRNA clusters in human, mouse and rat respectively. In our database, we have used a nomenclature for piRNA clusters. piRNA clusters are denoted by the chromosome number and the serial number of the cluster in that particular chromosome separated by a '.’. For example, '1.12’ denotes the 12^th^piRNA cluster in chromosome 1. Figure 5 shows the distribution of piRNA clusters obtained across entire human, mouse and rat genome. Chromosome 15 in human, chromosome 1 in both mouse and rat harbours the maximum number of piRNA clusters. We calculated the GC content of the piRNA clusters to measure their target binding stability within the genome. Since piRNAs are mostly derived from the repetitive regions of the genome (as mentioned earlier), so it is likely that the 'hotspots’ will also comprise of repeats. Hence, we mapped the chromosomal positions of these clusters with genes and repetitive regions of the genome.

Clusters can be either mono-directional or bi-directional. Mono-directional clusters have a single transcript whereas bi-directional clusters have two transcripts generated divergently from a common promoter. We thus curated the putative promoter regions obtained from MPromDB [34] and Li et al. [35] for the piRNA clusters residing in genes. This will give an overview of the putative promoters responsible for transcription of the piRNA cluster.

It has been reported that certain lncRNAs might serve as precursors of piRNAs and these lncRNAs in turn might be regulated by piRNAs [36]. Thus we have provided a user friendly genomic browser- piBROWSE (implemented using JBrowse 1.9.8 [37]) which will serve as a detailed guide showing the distribution of piRNAs and the relative presence of lncRNAs (lncRNA data obtained from Human BodyMap [38] and Ensembl Genes 74) (http://www.ensembl.org) across chromosomes. Besides this, piBROWSE also shows the distribution of piRNA occurrence relative to miRNAs (miRNA data obtained from miRBase [39]).

### Identifying motifs characterising piRNA clusters

As discussed above, piRNAs are known to originate from clusters but it is important to know whether there is any common sequence motif present within these clustered piRNAs. Hence, we determined *'characteristic motifs’* of the piRNA clusters using MEME [40] within the piRNAs constituting a cluster. As an input for MEME, the piRNA sequences present within the cluster were taken to determine the motifs (ranging in length from 6-32 nts). These motifs in a piRNA cluster could provide a clue towards designating piRNA clusters constituting piRNA families with their plausible common target binding sites.

### Exploring conserved piRNA clusters across species

It has been reported by Girard et al. [5] that piRNA clusters are conserved across mammalian species. We have looked for conservation of piRNA clusters across human, mouse and rat. We downloaded the entire set of human, mouse and rat syntenic regions from UCSC genome browser and mapped the piRNA clusters which are conserved. The annotated syntenic piRNA clusters can be visualized using the browser –piSynBrowse (implemented by modifying GSV [41]).

### Annotating pseudogene-associated piRNAs

**Figure 6:**
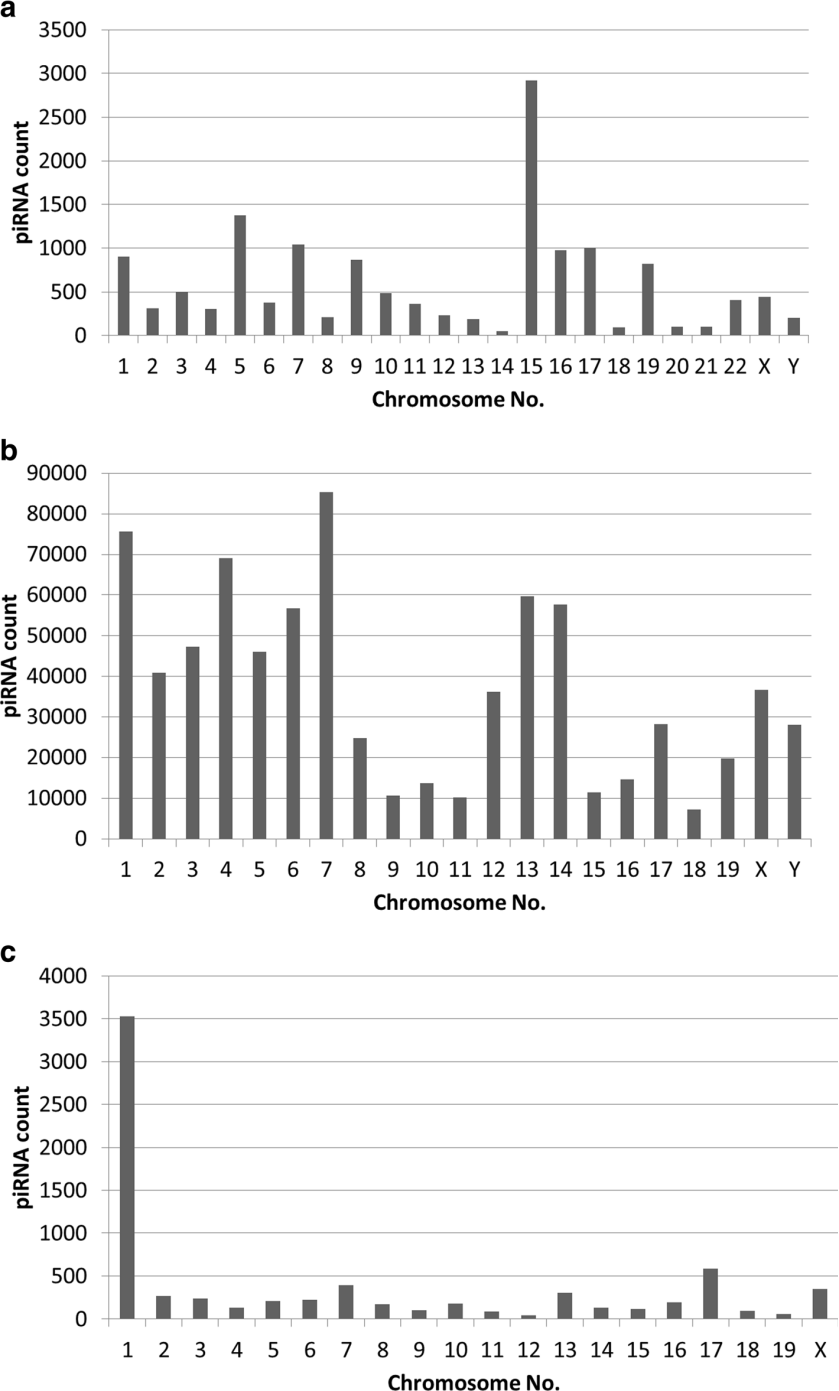
Distribution of pseudogene-associated piRNAs across (a) human genome (b) mouse genome and (c) rat genome.

Pseudogenes being non-functional counterpart of its parent gene were assumed to be “junk DNA”. Pseudogenes harbour the potential to regulate its functional counterpart gene. They do this with the help of small RNA mediated silencing. They are capable of producing siRNAs which in turn regulate the protein-coding genes. They also play an important role during cancer progression [18]. This prompted us to look for piRNAs residing in pseudogenes. Pesudogenes corresponding to human, mouse and rat genome were downloaded from http://www.pseudogene.org[42]. We obtained 16101, 18528 and 11054 pseudogenes for human, mouse and rat respectively. Using in-house perl scripts, we determined the localization of the piRNAs within pseudogenes using the corresponding coordinate information from http://www.pseudogene.org. Figure 6 shows the distribution of pseudogene-associated piRNAs across the entire human, mouse and rat genome. Majority of pseudogene-associated piRNAs were found in chromosome 15 of human, whereas for mouse and rat they were mostly found in chromosome 7 and 1 respectively.

### Analysing piRNA expression in different tissue and developmental stages

We used BLAST [31] and in-house perl scripts for analysing the expression profile of piRNAs in different tissues like testes, oocyte comprising different stages of their development. Users can view the analysis for each experiment which enlists 200 most abundant piRNAs along with their expression.

## Utility

### Search and output options

**Figure 7:**
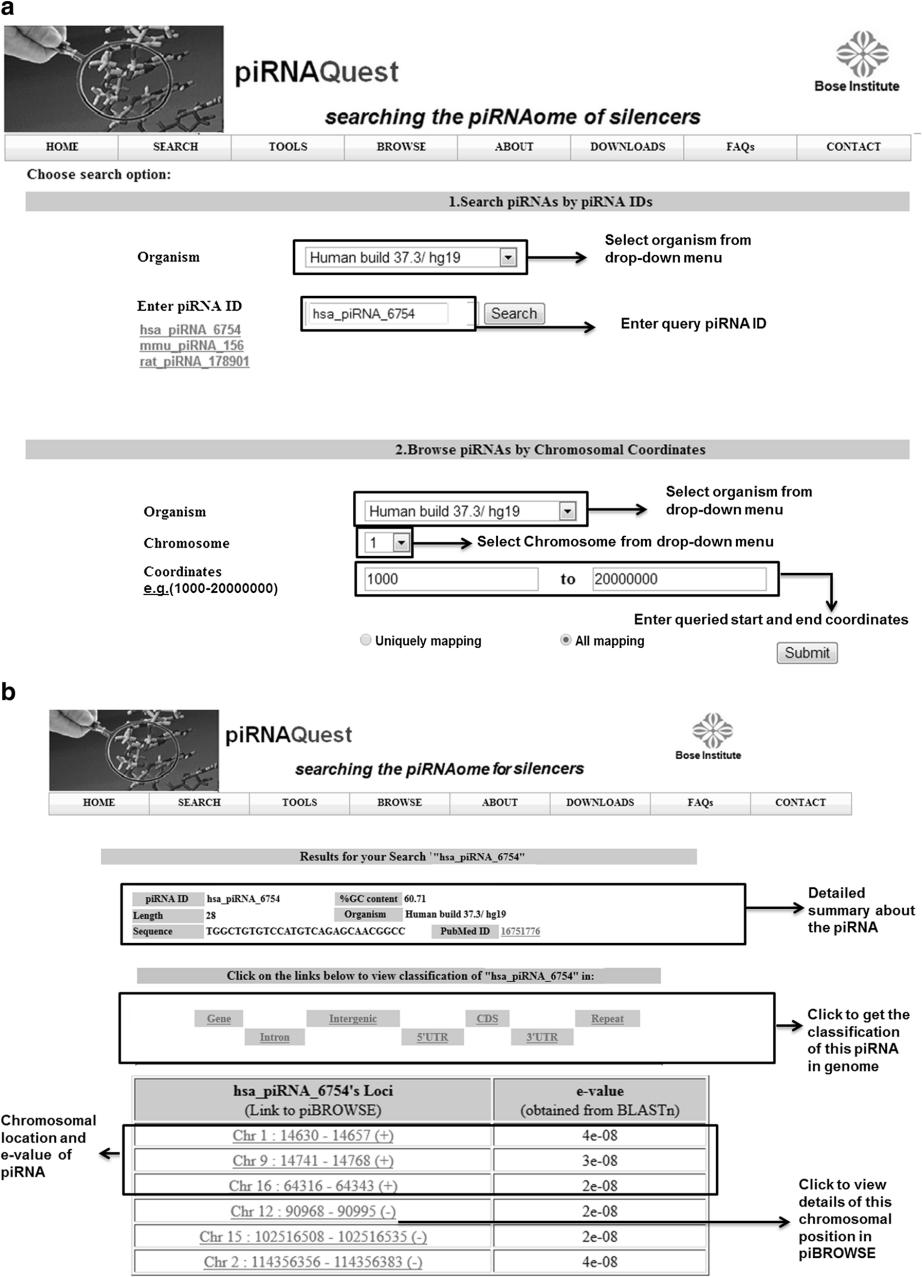
**Snapshot of “*search in piRNAs*” webpage showing the (a) search options and (b) the result page.** Users can search for the piRNA of interest by either entering piRNA ID or browse for the presence of piRNAs within a specific genomic loci. The result page displays the chromosomal coordinates and e-value of the corresponding alignment along with options to view its classification in the genome.

Search in piRNAs:

(a) *Search piRNAs by piRNA IDs:* users can search the database by piRNA ID for detailed information of the corresponding piRNA (sequence, length, %GC content, genomic location and classification in genes, intron, intergenic, CDS, 5^/^UTR, 3^/^UTR and repetitive elements). A snapshot of the search option along with its output is shown in Figure 7(a) and (b) respectively.

(b) *Browse piRNAs by chromosomal coordinates:* users can also retrieve detailed information of piRNAs by entering chromosomal coordinates. Moreover, users can also specifically browse for uniquely mapped piRNAs.

(c) *Browse Overlapping piRNAs:* users can also look into the Ping-Pong partners, repeat-associated overlapping piRNAs by defining the parameters-overlap distance, polarity and nucleotide bias.

Search piRNA in Genes:

(a) *Search piRNAs in Genes by Gene symbol/Gene Description/GO Term:* search for piRNAs overlapping in genes by gene symbol, gene description and GO term will retrieve the piRNAs corresponding to their localization in genes and their classification in intron, CDS, 5^/^UTR and 3^/^UTR respectively.

(b) *Browse piRNAs in Genes by chromosomal coordinates:* users can also retrieve detailed information of piRNAs in genes by entering chromosomal coordinates.

(c) *Browse piRNAs in pseudogenes by chromosomal coordinates:* users can retrieve detailed information of pseudogene-associated piRNAs and piRNA clusters by entering chromosomal coordinates.

Search piRNA in repeats:

(a) *Search repetitive piRNAs in Genomic Classifications:* users can search for repeats in 5^/^UTR piRNAs, 3^/^UTR piRNAs, CDS piRNAs, intronic piRNAs, intergenic piRNAs by either entering Repeat Family or Repeat Name.

(b) *Browse Repeat Associated piRNAs by Chromosomal Coordinates:* users can search for the repeat-associated piRNAs by defining the chromosomal coordinates and can also look for '*Ping-Pong*’ piRNAs, bias for 1U and 10A for '*Ping-Pong*’ piRNAs the present within that region.

Search piRNA in Cluster:

(a) *Browse piRNA Clusters by chromosomal coordinates:* users can search for piRNA clusters by entering chromosomal coordinates. This will retrieve information regarding cluster loci, cluster length, total piRNA within the cluster, prevalence of piRNA in plus/minus strand, %GC content in piRNA cluster and the corresponding motif. The link on the motif navigates to a page which help users to carry out further analysis of the motif obtained using MAST [43], FIMO [44] and GOMO [45].

(b) *Browse Genes in piRNA Clusters:* users can retrieve information of piRNA clusters overlapping with genes along with their putative promoters.

(c) *Browse Repeats in piRNA Clusters:* users can retrieve annotation of piRNA clusters overlapping with repeats.

Search stage specific piRNAs:

*Browse for Stage Specific piRNAs:* users can view the piRNAs associated with different developmental stages in human, mouse and rat.

*Search* syntenic piRNAs:

*Browse for Syntenic piRNA Clusters:* users can retrieve information of piRNA clusters overlapping with syntenic regions of human, mouse and rat by selecting the chromosome number for both target and query organism.

Output results for other search options as - *search piRNA in Genes, repeats, Cluster, syntenic regions* can be interpreted similarly as that for *search in piRNAs* as shown in Figure 7(b).

### Tools

#### *Homology with piRNAs*

Users can search for homology between their queried fasta sequences with the piRNAs annotated by piRNAQuest. This has been implemented using wwwblast-2.2.26 [31].

#### *Dynamic cluster*

A tool for determining the piRNA clusters (implemented on the basis of the definition provided by Lau et al. [4]) has been provided, where users can set parameters such as window increment, window size, minimum piRNA density, or any region of interest within the chromosome. This tool would find the piRNA clusters, the number of piRNAs within each cluster and their count in either strand using the user defined parameters. A cluster score will also be assigned to each output piRNA cluster.

#### *Pattern search*

This tool helps the user to search a specific sequence pattern or motif within the piRNA sequences annotated in the database. It reports the piRNA IDs which contain the user defined pattern. Users can use symbolic notations like * (which would mean any nucleotide in that particular position) and [NN] (showing the presence of either nucleotides in the position) to define the motif.

#### *Calculate AT-GC%*

This tool calculates the AT% and GC% for any given sequence(s) submitted in *.fasta* format.

### Browsing piRNAQuest

**Figure 8:**
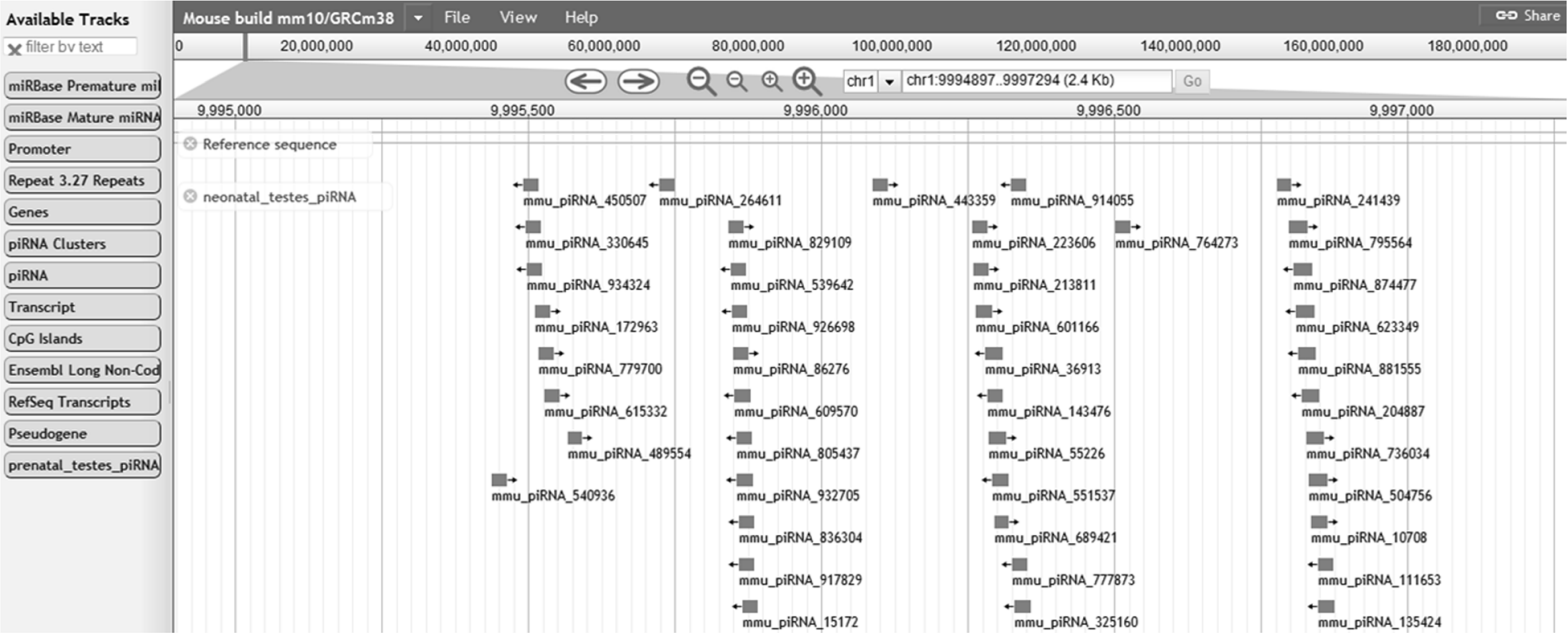
**Snapshot of piBROWSE showing the annotated piRNAs and piRNA clusters.** Shown on left, the tracks available for visualizing the annotations corresponding to the location of piRNA/piRNA cluster.

For viewing, the information related to piRNAs, a browser (piBROWSE) has been integrated. This has been implemented using JBrowse 1.9.8 [37]. This enables the user to visualize the chromosomal location with respect to piRNAs, lncRNA, premature and mature miRNAs, repetitive elements, genes, piRNA clusters, promoters and pseudogenes (as shown in Figure 8). Users can view selected tracks and navigate to any particular region of the genome. Stage specific piRNAs, Ping-Pong piRNA partners and uniquely mapped piRNAs can also be viewed.

**Figure 9:**
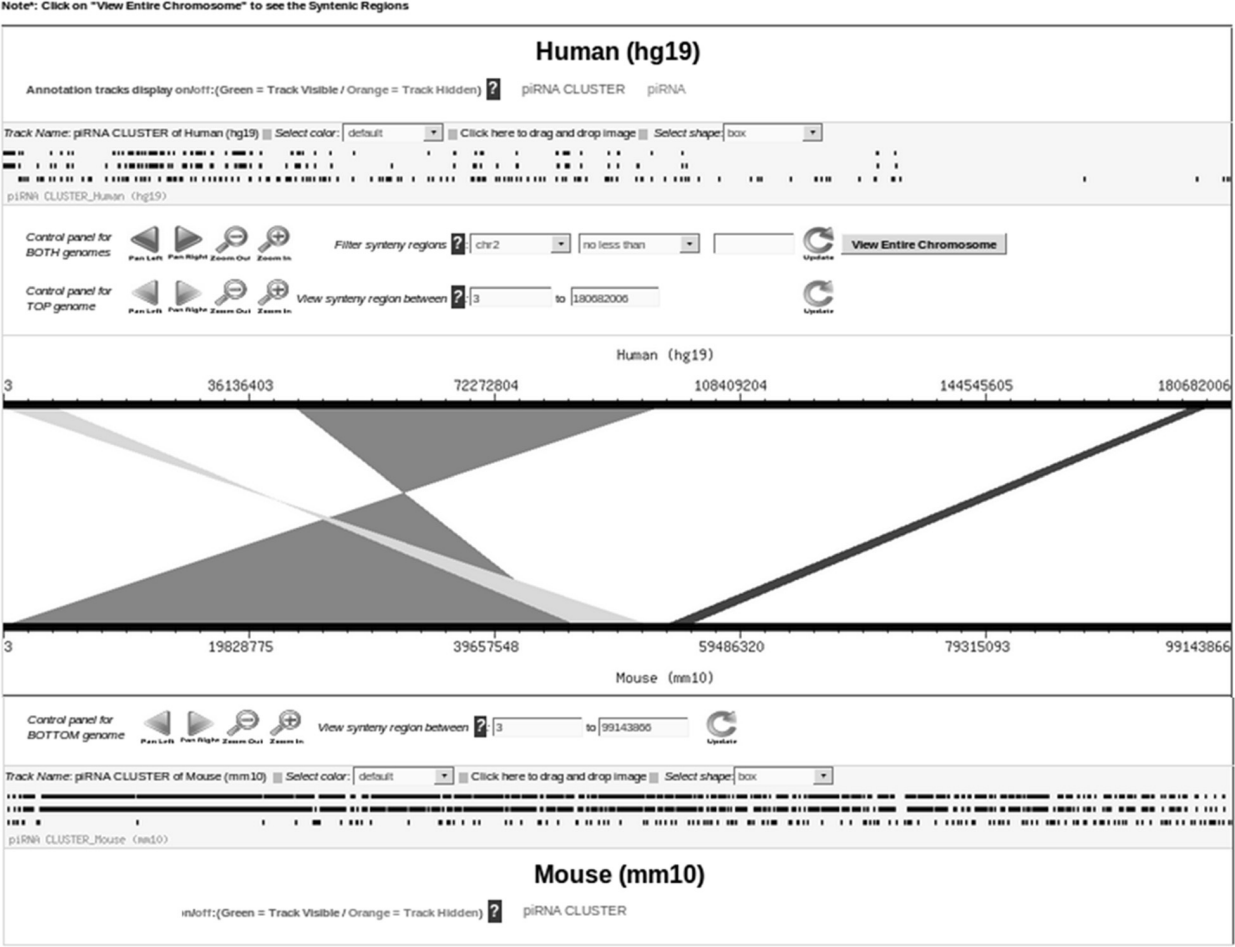
Snapshot of piSynBrowse showing the piRNA clusters corresponding to the syntenic regions in mouse with respect to human.

**Figure 10:**
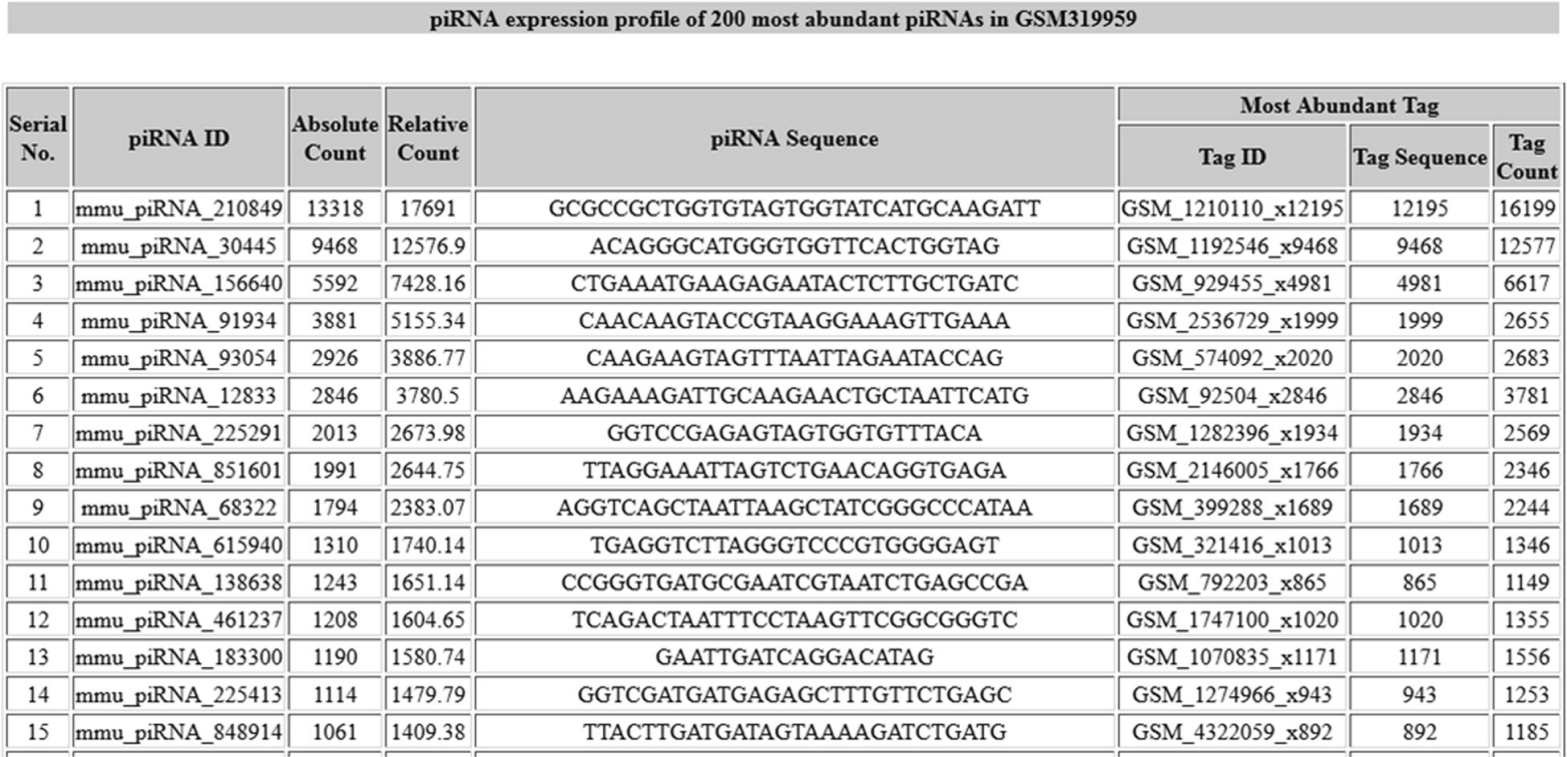
**Snapshot showing the expression profile of the most abundant piRNAs obtained on analysing GSM319959.** The data corresponds to 2dpp stage in mouse testes.

To view the interspecies conservation of the annotated piRNA clusters, the syntenic regions between human, mouse and rat can also be viewed (as shown in Figure 9). This has been implemented as piSynBrowse (modifying GSV [41]).Besides this, expression profile of piRNAs in different tissues like testes, oocyte comprising different stages of their development (16.5dpc, 2dpp, 10dpp, 30dpp, pachytene spermatocyte, round spermatid, adult etc.) obtained on analysis of small RNA sequence experiments has been provided within 'piRNA Expression’. Each analysis enlists the 200 most abundant piRNAs along with their expression profile (as shown in Figure 10).

## Discussion

piRNAs are revolutionizing the world of RNA interference. These act as 'guardian of the genome’ and are immensely important in regulating several vital processes like stem cell self-renewal. They are mostly known to protect the germline cells from the invasive transposable elements by piRNA-mediated gene silencing. This class of sncRNA has recently been found to act in non-gonadal cells as well. piRNAs are crucial in guiding several epigenetic mechanisms of the cell. Altogether, piRNAs are enigmatic and its complexity as well as uniqueness has made it a 'black-box’ which needs further exploration.

piRNAQuest is a unique and user friendly database that will serve as an enriched resource for piRNAs with respect to dataand information content in comparison to other existing databases. The key features of our database are classification of piRNAs based on their localization in gene, intron, intergenic, 5^/^UTR, 3^/^UTR, CDS and repetitive regions of the genome. We have also annotated piRNA clusters, pseudogene-associated piRNAs, 'characteristic motifs’ contained within clusters and promoters of the clusters residing within genes. Moreover a user-friendly browser has also been provided for easier and quick visualization of the detailed information regarding a particular piRNA.

piRNAs are widely distributed across the genome. Gan et al. [15] has reported that piRNAs in different stages of spermatogenesis i.e. spermatogonia A, round spermatid and pachytene spermatocytes map to introns, 3^/^UTR and CDSs but rarely map to 5^/^UTR region. Further, in Drosophila, it has been observed that piRNAs derived from genes preferentially arise from 3^/^UTR and are produced by a pathway that does not require the components of 'Ping-Pong’ biogenesis [46]. All these evidences hint towards the presence of genomic context dependent biogenesis and functionality of piRNAs. This prompted us to classify piRNAs with respect to their localization within CDS, 5^/^UTR, 3^/^UTR and intron. This classification will also help researchers gain an insight into the architecture of piRNA precursor transcript as well as their mode of biogenesis [15].

Besides this, we have classified intergenic piRNAs. More than 95% of piRNAs in adult mouse testes constitutes of intergenic piRNAs [35]. This instigated us to classify the intergenic piRNAs and gain a detailed overview of them. Figure 2 shows that intergenic piRNAs constitutes a major portion of human, mouse and rat piRNAs.As mentioned previously, piRNAs repress transposable elements thereby protecting the genome. These follow the 'Ping-Pong’ amplification cycle and silence transposons by cleaving their transcripts. Although it is assumed that piRNAs cleave these transposons (which comprises of repetitive elements) by searching for complementarity but how a particular piRNA specifically selects a transposable element still remains elusive. Hence it is important to have an idea regarding the distribution of repetitive elements in piRNAs. Thereby, we mapped the piRNAs to repeats (shown in Figure 3).

In addition to these, intergenic piRNAs which are devoid of transposable/repetitive elements have a different mode of biogenesis unlike the 'Ping-Pong’ biogenesis [46]. Emphasizing on this, we checked the piRNA abundance within the repetitive as well as non-repetitive regions contained in different genomic locations (CDS, 5^/^UTR, 3^/^UTR, intron and intergenic) within human, mouse and rat genome (Figure 4). Our observation reveals that major portion of intergenic piRNAs fall within the repetitive regions. A small portion of it falling within non-repetitive regions might be the candidate piRNAs whose biogenesis are independent of the 'Ping-Pong’ machinery as also observed by Gan et al. and Beyret et al. [15,17]. Further, there is a higher abundance of CDS piRNAs within non-repetitive regions of human, mouse and rat genome. This implies that not only a portion of intergenic piRNAs but other candidate piRNAs also have a possibility to follow a mode of biogenesis unlike the 'Ping-Pong’ mechanism. On the contrary, major intronicpiRNAs consist of repetitive elements.

Thereafter we annotated the hotspots of piRNA biogenesis i.e. piRNA clusters for human, mouse and rat respectively. There are certain loci in the genome from where piRNAs are generated in a clustered fashion. Most MILI- and MIWI-associated piRNAs are produced in a clustered manner. As can be seen from Figure 5, majority of the clusters are confined in chromosome 15 in human and chromosome 1 in mouse as well as rat. It has been reported in literature that some clusters contain one transcript (mono-directional) whereas others contain two transcripts (bi-directional) which are divergently transcribed from a common promoter region [17]. Thus we also scanned for the putative promoters of the piRNA clusters. This will help progress towards solving the unanswered queries on piRNA biogenesis and their mode of action. We checked for a *characteristic motif* present within each cluster which might provide a clue towards the target binding specificity of these clusters. We also looked for overlap of genes and repetitive elements within the piRNA clusters. Girard et al. [5] has mentioned that piRNA clusters are syntenic i.e. they maintain inter-species conservation. To explore this, we annotated the syntenic piRNA clusters between human, mouse and rat.

piRNAQuest provides another interesting feature wherein the piRNAs are mapped to pseudogenes thereby annotating pseudogene-associated piRNAs (as shown in Figure 6). Recent reports suggest that pseudogenes harbour ncRNAs which uses several fascinating mechanisms to control gene function [18]. For example, the Xist noncoding RNA, mediates dosage compensation and epigenetic repression by coating the inactive X chromosome in mammals. It has been reported that Xist thus evolved by pseudogenization of a protein-coding ancestor called Lnx3 [47]. In two other reports, it was shown in mouse oocytes, that portions of many pseudogene transcripts produce small interfering RNAs (siRNAs) [48,49]. Recently it has been reported that miRNAscoregulates gene-pseudogene pair [50]. Hence all these features make piRNAQuest a one-stop platform for studying and exploring the human, mouse and rat piRNAome.

### Future work

piRNAQuest will be updated regularly to incorporate more piRNA sequences and their annotations based on future experimental evidences. We further intend to analyse the motifs present in the piRNA clusters and classify them into plausible families as well as elucidating their mode of target binding.

## Conclusion

The various classifications and annotations curated in piRNAQuest will help researchers gain a better and in-depth insight into the evolving world of piRNAs. As discussed previously, piRNAs have the potential to regulate several vital processes of developmental biology as well as play a role in disease progression. Thus understanding the regulatory mechanism exhibited by them is very important. piRNAQuest will help towards developing a wider perspective regarding the biogenesis and functionality of this emerging class of sncRNA.

## Availability and requirements

piRNAQuest is freely accessible at http://bicresources.jcbose.ac.in/zhumur/pirnaquest/. It is best viewed in Mozilla Firefox browser with javascript enabled.

## Authors’ contributions

AS and RM: conception and design, collection and/or assembly of data, data analysis and interpretation, manuscript writing, SS: design and assembly of data, ZG: conception and design, data analysis and interpretation, drafting the manuscript and revising it critically for important intellectual content. All authors read and approved the final manuscript.

## Supplementary Material

Additional file 1: Table S1 Details of studies incorporated in piRNAQuest. Click here for file

Additional file 2: Figure S1 Workflow of piRNAQuest. Click here for file
