# Supplementary material for: piRNAQuest: searching the piRNAome for silencers
Source: BMC Genomics. 2014 Jul 4;15:555. doi: 10.1186/1471-2164-15-555 (PMC4227290; doi:10.1186/1471-2164-15-555)
Supplement: Additional file 1: Table S1 — Details of studies incorporated in piRNAQuest. [file 1471-2164-15-555-S1.doc]

| Sl. No | Organism | Tissue | Developmental stage | Protein involved | GSM ID | GSE ID | Pubmed ID | Group |
| --- | --- | --- | --- | --- | --- | --- | --- | --- |
| 1 | *Homo sapiens* | Testes | Adult | MIWI | - | - | 16751776 | Girard et.al |
| 2 | *Homo sapiens* | Epididymis | Adult | - | - | - | 22313525 | Yan Li et.al |
| 3 | *Homo sapiens* | Spermatozoa | Adult | - | GSM530234 | GSE21191 | 21989093 | Tagett R et al. |
| 4 | *Homo sapiens* | Spermatozoa | Adult | - | GSM530235 | GSE21191 | 21989093 | Tagett R et al. |
| 5 | *Mus musculus* | Testes | Adult | MIWI | - | - | 16751776 | Girard et.al |
| 6 | *Mus musculus* | Testes | Adult | - | - | - | 16778019 | Lau et.al |
| 7 | *Mus musculus* | Testes | 2dpp | - | GSM319959 | GSE12757 | 18922463 | Aravin et.al |
| 8 | *Mus musculus* | Testes | 10dpp | MILI | GSM319953 | GSE12757 | 18922463 | Aravin et.al |
| 9 | *Mus musculus* | Testes | 16.5dpc | MILI | GSM319956 | GSE12757 | 18922463 | Aravin et.al |
| 10 | *Mus musculus* | Ovary | 4-6 week old | - | GSM319958 | GSE12757 | 18922463 | Aravin et.al |
| 11 | *Mus musculus* | Testes | Pachytene spermatocyte | - | GSM610966 | GSE24822 | 21602304 | Gan et.al |
| 12 | *Mus musculus* | Testes | Round spermatid | - | GSM610967 | GSE24822 | 21602304 | Gan et.al |
| 13 | *Mus musculus* | Testes | 30dpp | - | GSM684484 | GSE27609 | 22842725 | Vourekas et.al |
| 14 | *Rattus norvegicus* | Testes | Adult | MIWI | - | - | 16751776 | Girard et.al |
| 15 | *Rattus norvegicus* | Testes | Adult | - | - | - | 16778019 | Lau et.al |

**Additional file 1: Table S1**
