# Supplementary figures and images for: piRNAQuest: searching the piRNAome for silencers
Source: BMC Genomics. 2014 Jul 4;15:555. doi: 10.1186/1471-2164-15-555 (PMC4227290; doi:10.1186/1471-2164-15-555)

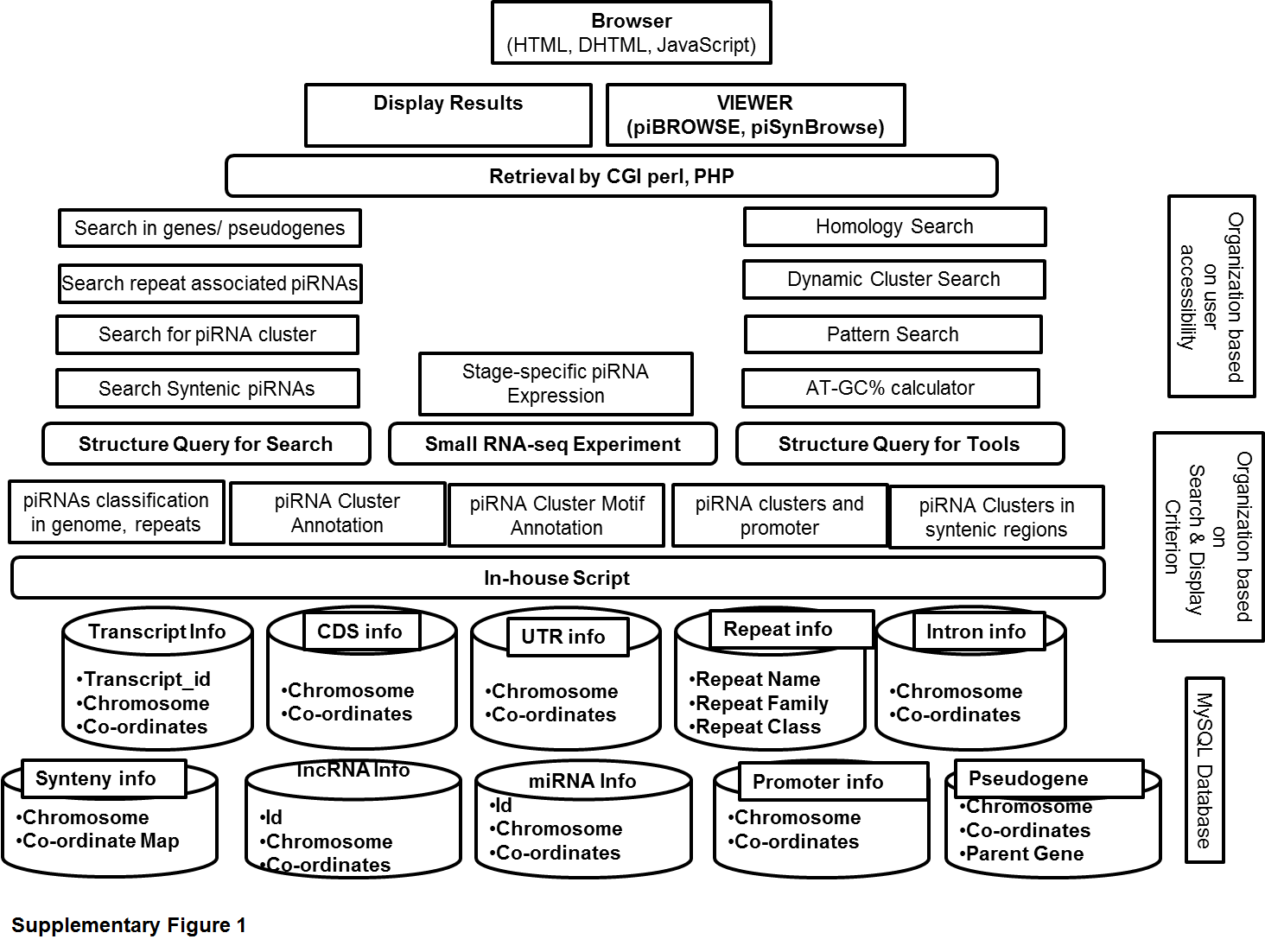

Supplement: Additional file 2: Figure S1 — Workflow of piRNAQuest. [file 1471-2164-15-555-S2.tiff]
